# The Prevalence of Dietary Supplement Use among College Students: A Nationwide Survey in Japan

**DOI:** 10.3390/nu9111250

**Published:** 2017-11-15

**Authors:** Etsuko Kobayashi, Yoko Sato, Keizo Umegaki, Tsuyoshi Chiba

**Affiliations:** Department of Food Function and Labeling, National Institute of Health and Nutrition, National Institutes of Biomedical Innovation, Health and Nutrition, 1-23-1 Toyama, Shinjuku-ku, Tokyo 162-8636, Japan; e-kbyshi@nibiohn.go.jp (E.K.); satoyoko@nibiohn.go.jp (Y.S.); umegaki@nibiohn.go.jp (K.U.)

**Keywords:** dietary supplements, college students, adverse effects, gender differences, Internet-based survey

## Abstract

To clarify the prevalence of dietary supplement use among college students, we conducted Internet-based nationwide questionnaire surveys with 157,595 Japanese college students aged between 18 to 24 years old who were registrants of Macromill Inc. (Tokyo, Japan). Among the 9066 respondents (response rate 5.8%), 16.8% were currently using dietary supplements. The prevalence of dietary supplement use did not differ significantly between males (17.1%) and females (16.7%). However, it increased according to their grade (13.1% to 20.5%), and it was higher in medical and pharmaceutical college students (22.0%) compared to others (16.7%). The main purpose of dietary supplement use was for the health benefits in both males and females. Other reasons were to build muscle in males, and as a beauty supplement and for weight loss in females. According to the purpose of dietary supplement use, the most commonly-used dietary supplements were vitamin/mineral supplements in both males and females, then protein and weight loss supplements in males and females, respectively. Although most students obtained information about dietary supplements via the Internet, they typically purchased the supplements from drug stores. Of the students surveyed, 7.5% who were currently using or used to use dietary supplements experienced adverse effects, with no significant difference between genders (8.8% in male, 7.0% in female). In conclusion, the prevalence of dietary supplement use increased with grade among college students in Japan. Some of them experienced adverse effects. Education may be important to prevent adverse effects resulting from supplement use in college.

## 1. Introduction

The prevalence of the use of dietary supplements, which are intended to provide the diet with additional nutrients, has globally increased; thus, a number of studies on this topic have been reported not only in Japan [1,2,3] but also in other countries such as the United States [4,5], and European countries [6]. Many of these studies have focused on the elderly population [7,8,9] and patients [10,11], who often use dietary supplements and frequently take medicines concomitantly. It was also reported by Sato et al. [12,13] that the prevalence of dietary supplement use among preschool children aged 0 to 6 years ranged from 8.0% to 15.5% in Japan, which is still lower than in the United States [14,15]. Children are given dietary supplements by their parents, mainly because parents believe that their children will not obtain sufficient nutrients from their diet. Parents tend to not only give their children vitamin/mineral supplements but also herbs or other ingredients in Japan [13]. The prevalence of dietary supplements in preschool children and junior/high school students most likely reflects their parents’ views. However, this situation may be different in college students, who seem to use them by their own free will.

The lifestyle of college students may differ from that of high school students. In Japan, many college students live alone upon admission to college and prepare their own meals. Some students may live with their family, but often do not regularly eat dinner with them because of other commitments such as extracurricular activities, part-time jobs, or dinner plans with fellow students. In addition, many college students skip breakfast [16,17]. These factors make them feel as if they are not getting enough nutrients from their diet, which might be a motivation to use dietary supplements for health benefits. Moreover, other factors might also lead to dietary supplement use in Japanese college students. Enticing information about dietary supplements is widespread from sources such as magazines, television commercials, and particularly the Internet. In addition, it is easy to purchase dietary supplements from drug stores, supermarkets, and convenience stores, as well as by mail order and via the Internet both domestically and overseas. Furthermore, prices vary for each product, even those that contain the same ingredients, thereby allowing individuals to choose the most reasonably priced dietary supplement.

There have been some reports about the prevalence of dietary supplement use in college students in Japan; however, these reports have just been university bulletins written in Japanese. Nonetheless, six independent studies reported that 16.6% or 49.3% (department of education), 29.7% or 32.0% (department of pharmacy), or 17.0% or 50.0% (department of nutrition) of college students use dietary supplements, respectively. However, the surveys were conducted in only one or a few colleges, which may be a reason for the wide range of prevalence shown in each study. The prevalence of dietary supplement use in students in other countries has also been reported, and was found to be 66.0% in the United States in 2015 [18] and 56.0% in Australia in 2015 [19]. In addition, studies have been conducted in athlete students, with 64.1% using supplements in the United States in 2013 [20] and 98.6% in Canada in 2005 [21]. Among medical students in the United States in 2005 [22] and pharmaceutical students in 2017 [23], almost half used dietary supplements. Similar to Japan, these studies were also conducted in only one or a few colleges. Currently, no nationwide survey of dietary supplement use in college students has been conducted in any country.

In this study, we conducted a nationwide survey using an Internet-based questionnaire to clarify the situation of dietary supplement use among college students.

## 2. Materials and Methods

### 2.1. Definition of “Dietary Supplement”

In Japan, a “dietary supplement” is not defined by law, and some dairy and soybean products are recognized as dietary supplements even if they are in the form of common foods. Therefore, the meaning of the term “dietary supplement” differs for each individual. In this survey, “dietary supplement” was defined as what college students considered to have beneficial effects on their health (e.g., vitamins, minerals, fish oil, amino acids, cereals) with the exception of food consumed daily (e.g., vegetables, fruit, milk, tofu).

### 2.2. Internet-Based Survey

An Internet-based questionnaire survey was conducted by Macromill Inc. (Tokyo, Japan) on their college student registrants (2 million, including 0.15 million college students in 2016) between 25 October and 7 November 2016. The Ministry of Education, Culture, Sports, Science and Technology reported that college students numbered approximately 2.7 million in Japan in 2016. This study was conducted with the approval of the Research Ethics Committee of the National Institutes of Biomedical Innovation, Health and Nutrition (No. 206-5, approved on 16 September 2016) and was in accordance with the Declaration of Helsinki. The questionnaire is given in the Supplementary Materials.

### 2.3. Preliminary Survey of Subjects Who Used Dietary Supplements

A preliminary survey (Table S1) was conducted with 157,595 college students aged between 18 and 24 years old that were registered on Macromill Inc. to select 10,000 respondents on a first-come, first-served basis. A total of 9066 college students (corresponding to 0.34% of college students in Japan) completed the preliminary survey after excluding inadequate responders (final response rate: 5.8%). The questionnaire included the prevalence of dietary supplement use and experience of education about food, nutrition, or dietary supplements at the college.

Students who were current or previous users of dietary supplements moved on to the full survey.

### 2.4. Full Survey

The full survey (Table S2) was conducted with 2745 students, and 2060 students completed the full survey (response rate: 75.0%). The questionnaire included the information source of dietary supplements, where they were obtained, the purpose of dietary supplement use (i.e., supplementation of nutrients, maintenance of health, improvements to health, beauty benefits, weight loss, building muscle, prevention of diseases, and treatment of disease), the type of dietary supplement used, any adverse effects experienced (e.g., diarrhea, constipation, stomachache, headache, nausea and vomiting, fatigue, eczema and itching, palpitations, worsening health upon examination), and, if yes, how they responded to the adverse effects.

### 2.5. Statistical Analysis

Differences among groups were evaluated using the χ^2^ test. Statistical analyses were performed using PASW statistics for Windows (version 18.0J, SPSS Inc., Chicago, IL, USA) and the level of significance was set at *p* < 0.05.

## 3. Results

### 3.1. Characteristics

The characteristics of the participants are shown in Table 1. The college students (*n* = 9066) who completed the preliminary survey were as follows: sex (males: 2966, females: 6100) and grade (1st year: 1838, 2nd year: 2132, 3rd year: 2068, 4th year: 2917, 5th year: 72, 6th year: 39). Those of students who completed the full survey (*n* = 2060) were as follows: sex (males: 565, females: 1495) and grade (1st year: 359, 2nd year: 430, 3rd year: 490, 4th year: 744, 5th year: 21, 6th year: 16). In addition, residential area is also shown in Table 1.

### 3.2. Prevalence of Dietary Supplement Use

The prevalence of dietary supplement use is shown in Table 2. In the preliminary survey, we asked whether the college students currently used or had previously used dietary supplements, and we received answers from 9066. Of these students, 1526 (16.8%) were currently using dietary supplements, 1375 (15.2%) had previously used them, and 6165 (68.0%) had never used them. The ratio of current users was similar between males (17.1%) and females (16.7%), but the prevalence of dietary supplement use significantly increased according to their grade (from 13.8% in 1st to 20.5% in 6th). In addition, the prevalence was 12.6% in junior college students, 16.7% in non-medical/non-pharmaceutical college students, and 22.0% in medical and pharmaceutical college students. The prevalence of dietary supplement use was also different by their level of dietary education. When they had learned about food and nutrition, the prevalence of dietary supplements was 19.9%, as opposed to 15.7% when they had not. When they had learned about dietary supplements, the prevalence was 21.9% versus 15.9%.

The full survey was conducted with 2060 college students who were using or had previously used dietary supplements.

### 3.3. Information Source and Ways to Obtain Dietary Supplements

The information sources of dietary supplement are shown in Table 3. The most popular way of obtaining information about dietary supplements was via the Internet (38.3%). This was followed by stores (33.6%), television (31.4%), family (25.3%), product labels (12.7%), friends or acquaintances (12.0%), pharmacists or drug store clerks (11.0%), newspapers, magazines, and flyers (10.4%). The ways that dietary supplements were obtained are shown in Table 4. Most students purchased them at pharmacies or drugstores (63.7%), followed by via the Internet (19.6%), from family members (14.8%), by mail order (12.1%), or from stores. There were some gender differences regarding information source and ways to obtain dietary supplements in that more males used the Internet as an information source and to purchase them compared to females. On the other hand, females were more dependent on their family members for information about dietary supplements.

### 3.4. Purposes of Dietary Supplement Use

The purposes of dietary supplement use are shown in Table 5. The main reason that college students used dietary supplements was to supplement their diet with nutrients (59.0%). Other reasons were maintenance of health (52.9%), beauty benefits (36.7%), weight loss (25.5%), improvements to health (15.4%), prevention of diseases (10.4%), and building muscle (9.6%). A small percentage of students used dietary supplements for treatment of disease (3.7%). There were gender differences regarding the purpose of dietary supplement use. Specifically, significantly more males used supplements for building muscle than females. On the other hand, significantly more females used them for beauty benefits and weight loss than males.

### 3.5. Types of Dietary Supplements Used

The types of dietary supplements used are shown in Table 6. Individual vitamins or minerals were more popular than a combination of multi-vitamins and minerals, or multi-vitamins or multi-minerals alone. Individual vitamins included vitamins B, C, and E; individual minerals included calcium, zinc, and iron. In females, 70.8% (179/253) of them used iron supplements. Furthermore, various non-vitamin and non-mineral supplements were also used by college students. Weight loss supplements (14.2%) were the most popular products, followed by protein/amino acids (8.4%), blueberry/lutein (6.2%), fish oil/n-3 polyunsaturated fatty acids (3.1%), and lactic bacterium products (2.3%). There are differences between males and females in non-vitamin, non-mineral supplements. The most popular dietary supplement was protein/amino acid supplements (23.7%) for males and weight loss supplements (18.1%) for females; blueberry/lutein supplements (8.3% in males, 5.4% in females) were the second most popular for both.

### 3.6. Adverse Effects

Adverse effects due to dietary supplement use are shown in Table 7. Of the students who completed the full survey (*n* = 2060), 7.5% (154/2060) experienced adverse effects, with a slightly higher prevalence in males (8.8%) than in females (7.0%). Among the adverse effects, diarrhea (33.8%) was the most frequent, followed by nausea and vomiting (25.3%), stomachache (24.0%), constipation (14.3%), and headache (12.3%). There were no differences in symptoms between males and females.

These symptoms may be associated with the use of dietary supplement categories. For example, diarrhea appears to be associated with using vitamin (42.3%) or weight loss supplements (32.7%); nausea and vomiting with minerals (20.5%), especially iron; and constipation with weight loss supplements (31.8%). The use of Lactobacilli-containing supplements was associated with abdominal pain. Weight loss supplements were reported to be associated with all of the symptoms we investigated, including constipation (31.8%). In addition, the number of dietary supplements that were used concomitantly was associated with more adverse effects. When students were using one product, the incidence rate of adverse effects was 4.9% (56/1140), when using two concurrently it was 8.9% (56/631); for three, 12.4% (25/202); for four, 21.6% (11/51); for five, 7.1% (1/14); and for more than six 22.7% (5/22).

### 3.7. Behavior after Experiencing Adverse Effects

It is important to inform public institutes of adverse effects related to dietary supplement use to prevent future cases. Therefore, we asked students what they did when they experienced adverse effects (Table 8). Almost half of the students (43.5%) immediately stopped using the dietary supplements. On the other hand, one-third of students (31.8%) did nothing. Some students consulted the people around them such as family members and friends (14.9%), or college mentors (7.8%). Only 6.5% of students reported the adverse effects to public institutes (1.9% to public health centers, 3.2% to the National Consumer Affairs Center of Japan or other consumer affairs center, and 3.2% to the Ministry of Health, Labor and Welfare or Consumer Affairs Agency, Government of Japan). More males consulted with or complained to others than females.

## 4. Discussion

We conducted a nationwide survey of the prevalence of dietary supplement use in college students, and found that 16.8% of the surveyed students were currently taking dietary supplements. The prevalence of dietary supplement use in college students in this survey is consistent with other surveys in Japan, but lower than in other countries [18,19,20,21,22,23]. The lower prevalence was also reported among preschool children in Japan [12,13] compared to the United States [14,15]. At this time, the prevalence of dietary supplement use in Japan might be lower than in other countries. In this study, students not only took vitamin/mineral supplements but also non-vitamin/non-mineral supplements for several purposes. There was no difference in the prevalence of use between males and females, but it increased according to their grade, suggesting that many students might begin taking dietary supplements in college.

The problem is that most college students do not understand the properties of dietary supplements or their ingredients, which are associated not only with beneficial effects but also with adverse reactions such as gastrointestinal and neurological complications, hepatotoxicity, and drug interactions [24,25,26,27]. It has been reported that adverse reactions from dietary supplements occur from infants to elderly people, with the highest frequency occurring in individuals aged 20–34. One study showed that about 25.5% of weight loss products (95% confidence interval [CI]: 23.1–27.9) and 10.0% of energy products (95% CI: 8.0–11.9) caused adverse effects, with 58.0% (95% CI: 52.2–63.7) of the reactions occurring in people 20 to 34 years of age in the United States [28]. In our study, 7.5% of college students who are currently using or used to use dietary supplements experienced adverse effects. In Japan, when consumers experience adverse effects, they have to report the incident to a public health center. However, only a few students (1.9%) reported to public health centers in this survey. At this time, we do not know the exact reasons, but it might be because the symptoms were not severe or they did not know that they had to report it.

Chatham-Stephens et al. analyzed 40 cases during April–December 2013, and reported that there were gender differences in the purposes and types of non-vitamin/non-mineral dietary supplements used in the United States [29]. More males used protein supplements for building muscle, whereas more females used dietary supplements for weight loss, which is consistent with our study. Adverse effects associated with these dietary supplements tend to occur in younger generations [29]. For example, with regard to the bodybuilding supplement OxyELITE Pro™ (USP labs LLC, Dallas, TX, USA), which contained 1,3-dimethylamylamine or aegeline, many people experienced adverse effects such as nausea, diaphoresis, cardiovascular disease, and hepatotoxicity when using OxyELITE Pro™ in the United States [29]. At this time, OxyELITE Pro™ is not marketed in Japan, but people can purchase it via the Internet. On the other hand, most young women want to lose weight to maintain their appearance, even though being excessively thin is a concern in this demographic in Japan [30,31]. Many ingredients used in weight loss supplements, such as *Coleus forskohlii*, red pepper, and senna, can cause diarrhea [32,33,34]. In fact, *Coleus forskohlii* extract (CFE) is one of the most popular ingredients in weight loss dietary supplements in Japan. Indeed, many female students used CFE supplements in this survey. However, many resultant adverse effects (mainly diarrhea) have been reported to public health institutes. Our group previously tried to determine the mechanism underlying CFE-induced diarrhea in a rodent model, but the rodents did not experience diarrhea even with excessive amounts of CFE. However, CFE induced hepatotoxicity, steatosis, and hepatic cytochrome P450 activation in rodents [35,36]. At this time, there have been no reports of CFE-associated hepatotoxicity in humans; however, precaution should be taken when using CFE supplements.

The recent trend in dietary supplement use in Japan was evident in this survey. Both male and female college students used blueberry/lutein supplements, which are believed to improve presbyopia, and used to be popular in elderly people. One reason is the “Food with Function Claims,” which is a new dietary supplement regulation that was implemented on April 2015 in Japan [37]. This new category of voluntary labeling allows companies to display a product’s specific health benefit (aka “functionality”) and an associated area of the human body on retail food packaging based on scientific evidence, but to claim about prevention or treatment of diseases is prohibit. With this new regulation, many blueberry/lutein supplements that claim to have benefits for the vision have hit the marketplace. Another reason dietary supplements have become so common is due to the popularity of smartphones in college students in Japan. In fact, several surveys have shown that more than 90% of college students use smartphones in Japan, which has been associated with an increase in symptoms of tired, burning, or itching eyes, and blurred vision, even in the young generation [38]. These symptoms are another reason that college students are attracted to dietary supplements with claims of eye benefits.

In this study, medical/pharmaceutical students (22.0%) were more likely to use dietary supplements than other college students (16.7%). There have been some reports of surveys conducted with medical students [22] and pharmaceutical students [23] in the United States, but these reports did not compare them to other college students. The higher preference of dietary supplement use in medical/pharmaceutical students might provide an opportunity to teach them about dietary supplements. However, each study [22,39] and systematic review [40] showed that the knowledge about dietary supplements is inadequate in medical/pharmaceutical students as well as in physicians/pharmacists, because they might learn about dietary supplements generally, but not about the actual status of them, such as their evidence levels of efficacy, quality of products, and adverse events. Appropriate knowledge could help clinicians who have consultations with patients who take dietary supplements. Most patients do not inform their physicians/pharmacists about their dietary supplement use, and communication between patients and physicians/pharmacists is important for preventing adverse effects not only in Japan [11] but also in the United States [41]. In addition, the prevalence of dietary supplement use was different in terms of knowledge about food, nutrition and dietary supplements: in this study it was higher in students who learned about food, nutrition, and dietary supplements. At this time, a cause–effect relationship is not clear, but education should promote diet quality and suppress dietary supplement use, especially in this generation. Thus, more education on dietary supplements is needed for college students, especially medical/pharmaceutical students, which would not only guide their own use but also serve their future patients well.

There were some limitations in this study. First, this survey was conducted using an Internet-based questionnaire. The Ministry of Internal Affairs and Communications reported that more than 98% of Japanese college students used the Internet in 2015, but only 20% of college students have experience with responding to Internet-based survey. In this study, the number of respondents was limited in the preliminary survey and the full survey (0.34% and 0.08% of college students in Japan, respectively). The male: female ratio was different between the general college population and our respondents. It was 1:0.75 in the general Japanese college population, versus 1:2 in our preliminary survey and 1:3 in the full survey. This difference might be caused by the ratio of registrants in Macromill Inc., which was 1:2 in teenagers and twenties. However, there was no gender difference in the prevalence of dietary supplement use and incidence of adverse effects. In addition, we surveyed students aged between 18 and 24 years old, but 0.14% of the general college population is older than 25. If we include those 25 years and older, the prevalence of dietary supplement use might increase. So, our respondents were a convenience sample of Japanese college students. However, the respondents included both sexes, several grades, each residential area, and a variety of departments and colleges. Second, we did not conduct a Food Frequency Questionnaire on these respondents, so we could not evaluate whether they gained enough nutrients from their diet, and whether taking vitamin/mineral supplements were needed for their health. Furthermore, some Japanese consumers, even college students, do not understand the difference between dietary supplements and medicines. Indeed, some students wrote the names of medicines instead of dietary supplements in this study; others noted just the main ingredient of the dietary supplement, even though we asked for the name of the product. Most products contain several ingredients, so efficacy and safety should be evaluated for the whole products, not for individual ingredients. This indicates that some students may not understand the difference between ingredients and final products.

## 5. Conclusions

In this survey, we explored the prevalence of dietary supplements in college students in Japan, since a proportion of them start using dietary supplements while in college. However, some of them used dietary supplements inappropriately, expecting them to treat disease or using several products concomitantly. Education about food, nutrition, and dietary supplements could not suppress dietary supplement use. In addition, some of them experienced adverse effects. Therefore, college students need to be better educated about dietary supplements.

## Figures and Tables

**Table 1:** Characteristics of surveyed students.

	Preliminary Survey		Full Survey	
	*n*	%	*n*	%
All	9066		2060	
**Sex**				
Male	2966	32.7	565	27.4
Female	6100	67.3	1495	72.6
**Grade ^1^**				
1st	1838	20.3	359	17.4
2nd	2132	23.5	430	20.9
3rd	2068	22.8	490	23.8
4th	2917	32.2	744	36.1
5th	72	0.8	21	1.0
6th	39	0.4	16	0.8
Residential area				
Hokkaido	318	3.5	80	3.9
Tohoku	513	5.7	133	6.5
Kanto	3483	38.4	780	37.9
Chubu	1310	14.4	283	13.7
Kinki	1899	20.9	425	20.6
Chugoku	462	5.1	105	5.1
Shikoku	192	2.1	38	1.8
Kyusyu	889	9.8	216	10.5

^1^ Junior college (1st–3rd), college (1st–4th), medical and pharmaceutical college (1st–6th).

**Table 2:** Prevalence of dietary supplement use.

	*n*	Currently Use	Previously Used	Never Used	*p*-Value
All	9066	16.8	15.2	68.0	
**Sex**					<0.001
Male	2966	17.1	12.3	70.6	
Female	6100	16.7	16.6	66.7	
**Grade**					<0.001
1st	1838	13.8	13.1	73.1	
2nd	2132	15.8	15.1	69.2	
3rd	2068	17.8	15.9	66.3	
4th	2917	18.7	15.8	65.5	
5th	72	20.8	18.1	61.1	
6th	39	20.5	25.6	53.8	

Statistical analyses were conducted among gender or grade. *p*-values were calculated using the χ^2^ test.

**Table 3:** How do you get information about dietary supplements? (%).

	All (2060)	Male (565)	Female (1495)	*p*-Value
Internet	38.3	45.1	35.7	<0.001
Stores	33.6	29.7	35.1	0.021
Television	31.4	31.7	31.3	0.869
Family	25.3	17.7	28.2	<0.001
Product labels	12.7	12.0	12.9	0.595
Friends or acquaintances	12.0	14.5	11.0	0.030
Pharmacists or drug store clerks	11.0	10.1	11.4	0.407
Newspapers, magazines, flyers	10.4	7.8	11.4	0.017
Clinic (physicians, pharmacists, dietitians)	2.8	3.4	2.5	0.311
Radio	2.3	5.1	1.2	<0.001
Inquire manufacturer	1.0	2.7	0.4	<0.001
Others	1.2	1.8	1.0	0.156

Multiple answers. Statistical analyses were conducted between males and females. *p*-values were calculated using the χ^2^ test.

**Table 4:** How do you obtain dietary supplements? (%).

	All (2060)	Male (565)	Female (1495)	*p*-Value
Pharmacy or drugstore	63.7	56.3	66.6	<0.001
Internet	19.6	28.5	16.3	<0.001
Family bought	14.8	12.0	15.8	0.032
Mail order	12.1	13.1	11.8	0.411
Supermarket	8.8	11.7	7.8	<0.001
Convenience store	6.4	10.3	4.9	<0.001
Retail store	5.5	9.4	4.1	<0.001
Department store	3.0	3.9	2.7	0.149
Friends or acquaintances	1.8	2.8	1.4	0.030
Co-op store	1.1	1.9	0.8	0.027
Others	1.6	1.9	1.5	0.443

Co-op: consumers’ cooperative. Multiple answers. Statistical analyses were conducted between male and female. *p*-values were calculated using the χ^2^ test.

**Table 5:** What is the purpose of dietary supplement use? (%).

	All (2060)	Male (565)	Female (1495)	*p*-Value
Supplementation of nutrients	59.0	61.9	57.9	0.098
Maintenance of health	52.9	59.5	50.4	<0.001
Beauty benefits	36.7	12.9	45.6	<0.001
Weight loss	25.5	12.4	30.5	<0.001
Improvements to health	15.4	16.6	14.9	0.334
Prevention of diseases	10.4	12.7	9.6	0.035
Building muscle	9.6	28.0	2.7	<0.001
Treatment of diseases	3.7	4.6	3.4	0.204
Others	1.7	3.2	1.1	0.001

Multiple answers. Statistical analyses were conducted between male and female. *p*-values were calculated using the χ^2^ test.

**Table 6:** Which kind of dietary supplements are you using or did you use? *n* (%).

	All (2060)	Male (565)	Female (1495)	*p*-Value
**Vitamin/Mineral**				
Multi-vitamins and minerals	43 (2.1)	15 (2.7)	28 (1.9)	0.268
Multi-vitamins	178 (8.6)	71 (12.6)	107 (7.2)	<0.001
Multi-minerals	24 (1.2)	9 (1.6)	15 (1.0)	0.266
Individual vitamin	472 (22.9)	86 (15.2)	386 (25.8)	<0.001
Individual mineral	366 (17.8)	113 (20.0)	253 (16.9)	0.103
Any type	1083 (52.6)	294 (52.0)	789 (52.8)	0.764
**Non-Vitamin, Non-Mineral (Top 5)**				
Weight loss	293 (14.2)	22 (3.9)	271 (18.1)	<0.001
Protein/Amino acid	174 (8.4)	134 (23.7)	40 (2.7)	<0.001
Blueberry/Lutein	128 (6.2)	47 (8.3)	81 (5.4)	0.015
Fish oil/*n*-3 PUFA	63 (3.1)	21 (3.7)	42 (2.8)	0.286
Lactic bacterium	47 (2.3)	10 (1.8)	37 (2.5)	0.339

PUFA: polyunsaturated fatty acids, Multiple answers. Statistical analyses were conducted between male and female. *p*-values were calculated using the χ^2^ test.

**Table 7:** Have you ever experienced adverse effects due to dietary supplement use? If yes, what symptom(s) did you experience? (%).

	All (2060)	Male (565)	Female (1495)	*p*-Value
Never	92.5	91.2	93.0	0.145
Yes	7.5	8.8	7.0	
**Symptom ^1^**				
Diarrhea	33.8	32.0	34.6	0.748
Nausea and Vomiting	25.3	30.0	23.1	0.355
Stomachache	24.0	28.0	22.1	0.424
Constipation	14.3	16.0	13.5	0.673
Headache	12.3	16.0	10.6	0.338
Eczema and Itching	10.4	14.0	8.7	0.309
Fatigue	7.8	10.0	6.7	-
Palpitations	3.9	6.0	2.9	-
Results of medical check had become worse	1.9	2.0	1.9	-
Others	6.5	0.0	9.6	-

^1^ All (*n* = 154), Male (*n* = 50), Female (*n* = 104). Multiple answers. Statistical analyses were conducted between male and female. *p*-values were calculated using the χ^2^ test.

**Table 8:** How did you responses to the adverse effects? (%).

	All (154)	Male (50)	Female (104)	*p*-Value
Stopped using dietary supplements immediately	43.5	34.0	48.1	0.099
Did nothing	31.8	16.0	39.4	0.003
Consulted with family or friends	14.9	32.0	6.7	<0.001
Consulted with mentors in college	7.8	18.0	2.9	-
Complained to manufacturer	7.1	16.0	2.9	-
Complained to the retail store	5.2	14.0	1.0	-
Went to a hospital	5.2	4.0	5.8	-
Reported the incident to the National Consumer Affairs Center of Japan or other consumer affairs center	3.2	6.0	1.9	-
Reported the incident to the MHLW or Consumer Affairs Agency, Government of Japan	3.2	10.0	0.0	-
Reported the incident to public health center	1.9	6.0	0.0	-
Other	2.6	6.0	1.0	-

MHLW: Ministry of Health, Labor and Welfare, Multiple answers. Statistical analyses were conducted between male and female. *p*-values were calculated using the χ^2^ test.

## Supplementary Materials

The following are available online at www.mdpi.com/2072-6643/9/11/1250/s1, Table S1: Preliminary Survey, Table S2: Full Survey.
