# Supplementary material for: The Prevalence of Dietary Supplement Use among College Students: A Nationwide Survey in Japan
Source: Nutrients. 2017 Nov 15;9(11):1250. doi: 10.3390/nu9111250 (PMC5707722; doi:10.3390/nu9111250)
Supplement: Supplementary file 1 [file nutrients-09-01250-s001.pdf]

Table S1. Preliminary Survey

Q1: Have you ever learned about food or nutrition in your college?
A1: Yes, I have.
A2: Not yet. But, I will.
A3: No, I have not. And I will not.
A4: I don't remember it.
**Q2: Have you ever learned about dietary supplements in your college?**
A1: Yes, I have.
A2: Not yet. But, I will.
A3: No, I have not. And I will not.
A4: I don't remember it.
**Q3: Have you ever used dietary supplements?**
A1: Yes, I am using dietary supplements.
A2: Yes. I used to use dietary supplements, but I quit.
A3: No, I have never used them.

Note: A segment of the people who answered "A1 or A2 in Q3" moved on to the full survey.

Table S2. Full Survey

Q1: How do you get information about dietary supplements? – Multiple choice
A1: Television
A2: Radio
A3: Newspapers, magazines, flyers
A4: Internet
A5: Stores
A6: Clinic (physicians, pharmacists, dietitians)
A7: Pharmacists or drug store clerks
A8: Manufacturer
A9: Product labels
A10: Family
A11: Friends or acquaintances
A12: Other
**Q2: How do you obtain dietary supplements? – Multiple choice**
A1: Pharmacy or drugstore
A2: Retail store
A3: Convenience store
A4: Supermarket
A5: Department store

A6: Internet<br>A7: Mail order<br>A8: Co-op store<br>A9: Family<br>A10: Friends or acquaintances<br>A11: Other
**Q3: What is the purpose of dietary supplement use? – Multiple choice**
A1: Maintenance of health<br>A2: Supplementation of nutrients<br>A3: Beauty benefits<br>A4: Weight loss<br>A5: Building muscle<br>A6: Improvement of health<br>A7: Prevention of diseases<br>A8: Treatment of diseases<br>A9: Other
**Q4: How many dietary supplements are you using (or did you use)?**
A1: 1<br>A2: 2<br>A3: 3<br>A4: 4<br>A5: 5<br>A6: 6+
**Q5: Which dietary supplements are you using or did you use? – Free answer, up to 5 products**
**Q6: Have you ever experienced adverse effects due to dietary supplement use?**<br>**If yes, what symptom(s) did you experience? – Multiple choice**
A1: No, I have never experienced adverse effects.<br>A2: Nausea and vomiting<br>A3: Headache<br>A4: Stomachache<br>A5: Diarrhea<br>A6: Constipation<br>A7: Eczema and Itching<br>A8: Fatigue<br>A9: Palpitations<br>A10: Results of medical check had become worse<br>A11: Other
**Q7: How did you response to adverse effects? – Multiple choice**

A1: Did nothing

A2: Stopped using dietary supplements immediately

A3: Consulted with family or friends

A4: Consulted with mentors in college

A5: Complained to manufacturer

A6: Complained to the retail store

A7: Reported to the National Consumer Affairs Center of Japan or other consumer affairs center

A8: Reported to the Ministry of Health, Labor, and Welfare or Consumer Affairs Agency, Government of Japan

A9: Reported to public health center

A10: Went to a hospital

A11: Other
